# Red and blue photo-selective nets optimize leaf photostructure and photosynthetic efficiency to enhance antioxidant capacity, yield, and quality in adzuki bean

**DOI:** 10.3389/fpls.2025.1670702

**Published:** 2025-11-03

**Authors:** Congpei Yin, Zhaojin Shi, Dongxiao Li, Cheng Tian, Yuancong Li, Weixin Dong, Yuechen Zhang

**Affiliations:** ^1^ State Key Laboratory of North China Crop Improvement and Regulation/Key Laboratory of Crop Growth Regulation of Hebei Province/College of Agronomy, Hebei Agricultural University, Baoding, China; ^2^ College of Agronomy and Medical, Hebei Open University, Shijiazhuang, China

**Keywords:** adzuki bean (*Vigna angularis* L.), photo-selective nets, photosynthetic physiology, antioxidant properties, yield, quality

## Abstract

Adzuki bean (*Vigna angularis* L.) is a characteristic economic crop with ecological adaptability and industrial potential. During crop production and cultivation, regulating light quality through photo-selective nets or films has become an important environmental control strategy for optimizing their yield and quality. This experiment studied the effects of different color photo-selective nets (red photo-selective net, RN; blue, BN; yellow, YN; green, GN; natural light, CK) on the yield, quality, agronomic traits, photosynthetic characteristics and antioxidant capacity of adzuki beans. The results demonstrate that, compared with CK, RN treatment significantly increased plant height (12.23%) and total dry weight (18.04%), while BN treatment significantly enhanced stem diameter (8.15%), root dry weight (21.62%) and root-shoot ratio (23.53%). RN and BN treatments both optimized the anatomical structure of leaves, showing that palisade tissue and spongy tissue increased significantly by 8.68%, 49.74% and 21.01%, 66.96% respectively compared with CK. This is possibly related to the increased net photosynthetic rate (RN: 23.18% and BN: 14.44% higher than CK) and stomatal conductance (RN: 15.82% and BN: 21.65% higher than CK) to minimize photosynthetic “noon break” depression, and ultimately enhancing the light energy conversion efficiency and actual light energy capture efficiency of the PSII reaction center. Under both RN and BN treatments, the activities of antioxidant enzymes (SOD, POD, CAT) were enhanced to suppress reactive oxygen species (ROS) accumulation; Meanwhile, the malondialdehyde (MDA) content was correspondingly reduced by 11.62% and 39.08% compared to CK. Crucially, RN treatment significantly enhanced the yield of adzuki beans (15.05%), starch content (4.69%), and total phenol content (20.00%) compared to CK. BN treatment substantially increased yield (10.63%), soluble protein content (7.69%), amino acid content (9.55%), and total flavonoid content (38.05%). Under GN treatment, although the yield of adzuki beans decreased (8.56%) and the net photosynthetic rate reduced (2.25%), the total flavonoid content of adzuki beans significantly increased by 51.21%. In conclusion, the red and blue light treatments enhance both photosynthetic capacity and yield and improve quality traits in adzuki beans, offering novel insights into optimizing light environments in cultivating specialized legume varieties.

## Introduction

Adzuki bean (*Vigna angularis* L.) is a characteristic crop sharing both ecological adaptability and industrial potential. Its seeds are enriched in starch and plant protein, which can be converted into bioethanol, degradable materials and protein-based products (Ren et al., 2021). The stem and pod fibers can be processed into nanocomposites or biochar for energy and industrial purpose (Remanan and Zhu, 2020). Antioxidant components (anthocyanins, polyphenols, etc.) can be further developed into natural preservatives, biopesticides and cosmetic raw materials. Adzuki beans have the characteristics of tolerating poor soil and nitrogen fixation, which can support the sustainable planting on marginal land and promote the development of green agriculture and circular economy (Li et al., 2021; Lin et al., 2021).

Light critically regulates plant growth and photosynthetic processes (Chen et al., 2021). Light parameters-including intensity, spectral quality, and photoperiod-modulate the plant development by triggering physiological responses and secondary metabolisms (Fan et al., 2019). Under optimal conditions, enhanced light-harvesting and electron transport efficiency improve carbon dioxide (CO_2_) fixation and net photosynthetic rates (Yang et al., 2018). Conversely, excessive irradiance causes photoinhibition and photooxidative damage for photosynthetic tissues (Wang et al., 2018).

The ongoing global warming trend poses severe challenges to the agricultural production. Key associated challenges encompass elevated atmospheric CO_2_ concentration, raised air temperature (AT), intensified solar radiation (Hu et al., 2018), reduced frequency of frost days, increased occurrence of tropical nights (Howden et al., 2007), and scarce water resources (Zhou et al., 2018). Excess photosynthetically active radiation (PAR) induces photoinhibition, intensified heat stress and stomatal closure, consequently reducing net photosynthesis and limiting the primary process for growth. Consequently, strategic regulation of crop environment has emerged as a key technology for enhancing the yield and quality of medicinal and edible plants (Miao et al., 2016). Promoted by the evolving agricultural practices, particularly the advancement of facility agriculture, photo-selective shade netting has gained global adoption. This technology serves as dual functions: modulating both spectral composition (quality) and intensity (quantity) of incident light, while concurrently improving crop yield, quality, and phytochemical profiles. Additionally, it provides crop protection against adverse environmental conditions (Abbasnia Zoare et al., 2019).

Photo-selective shade nets are used as an effective technique for large scale manipulation of the spectral proportion of light in crop environments. Unlike conventional shade nets, the photo-selective nets incorporate light-dispersing and light-reflecting chromatic additives during manufacturing. Consequently, the incident light quality (spectral composition) is altered upon transmission through the netting. These nets then modulate both light quality and quantity by increasing the proportion of diffuse light (scattered) (Shahak, 2008) and selectively enhancing absorbing specific spectral bands (Selahle et al., 2015). Furthermore, they influence the microclimate factors, including airflow, temperature and humidity.

Extensive research has demonstrated that photo-selective nets of different colors elicit distinct morphological and physiological responses in plants, and that these responses exhibit significant inter-specific variation (Lusk and del Pozo, 2002). For instance, red and black photo-selective nets promote plant height and canopy expansion in *M. africana*, whereas blue shade nets tend to suppress stem elongation. Similarly, studies on lettuce (*Lactuca sativa*) have revealed that the plants grown under blue or black photo-selective nets typically accumulate the highest contents of leaf total chlorophyll compared to those under nets of other colors (Ilić et al., 2017). At maturity, the carotenoid contents were increased in piquin peppers treated with blue photo-selective net (Viveros and Banuet, 2023). Blue nets enhanced plant yield compared to black nets in tea plants (Jin et al., 2021), while red net shading resulted in significantly higher yield than black net shading in sweet pepper (Darázsi, 2014). Compared to unshaded control, the black, blue, and red nets enhanced plant growth index, net photosynthetic rate (Pn), and stomatal conductance (Gs) in *Camellia sinensis* (Zhang et al., 2022). Furthermore, in *Myrsine africana*, the red, black and blue nets reduced starch and sucrose contents, likely attributable to its lowered photosynthetically active radiation (PAR) levels, reduced light intensity, and elevated blue light fraction (Coles et al., 2021).

Despite extensive research on light quality regulation for crop growth, predominantly focused on horticultural crops was conducted, the effects of photo-selective nets on photosynthetic characteristics, yield, and quality remain unreported in adzuki beans (*Vigna angularis* L.). Therefore, elucidating the spectral-response mechanisms in adzuki bean is critical for developing the tailored cultivation strategies that enhance productivity and resource-use efficiency within its production systems. The distinct light environments generated by different photo-selective net treatments were hypothesized to influence the phenotypic traits of adzuki bean. The study investigated photosynthetic physiological characteristics, yield, quality and antioxidant capacity in this legume species. It aimed to provide an in-depth understanding of the effects of different spectral conditions on plant growth and photosynthetic mechanisms, thereby establishing a theoretical foundation for achieving steady yield and quality in adzuki bean.

## Materials and methods

### Plant material and sample preparation

**Figure 1:**
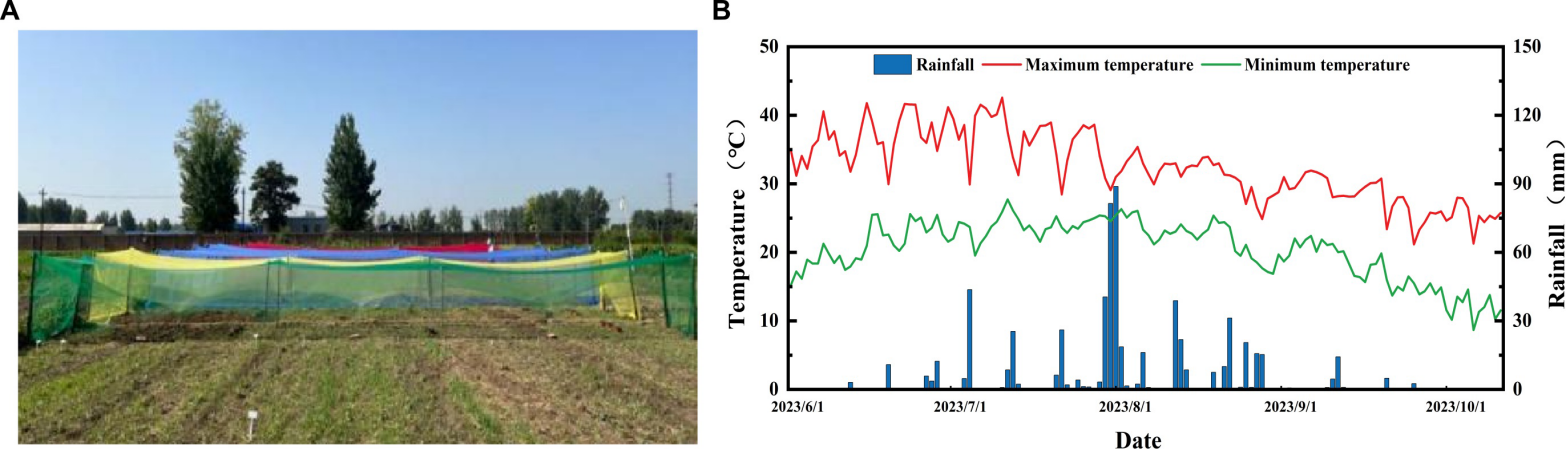
**(A)** Photographs of different photo-selective net treatments. **(B)** Air temperature and rainfall amount during adzuki bean planting in 2023.

The experiment was carried out at the experimental base of Hebei Agricultural University (N38°87’, E115°47’) in Baoding City, Hebei Province in 2023. The late-maturing and photoperiod-sensitive adzuki bean (*Vigna angularis* L.) variety ‘Jihong 16’ was provided by the Grain and Oil Crop Research Institute, Hebei Academy of Agriculture and Forestry Sciences. The seeds were sown on June 26, with a row spacing of 50 cm and a plant spacing of 25 cm. Plants were cultivated under photo-selective nets using a tent-covering method. Specifically, the nets (Fuzhou Kangsheng Knitting Textile Co., Ltd., China) were installed on the upper part of support structure when plants developed two fully-expanded true leaves. The support structures constructed were 1 m in height, 2 m in width, and 15 m in length. The experiment comprised five treatments, including a control (CK) under natural light and four treatments established by photo-selective nets of different colors: red (RN), blue (BN), green (GN) and yellow (YN). A completely randomized design (CRD) was employed with three biological replicates per treatment. Photosynthetically active radiation (PAR) beneath each photo-selective net and under control (CK) was recorded using HOBO data loggers (Onset Computer Corporation, Bourne, MA, USA), which was to be used to calculate the shading level=PAR_NET_/PAR_CK_, with the shading level of photo-selective nets of 15 ± 3% (**Table 1**; **Figure 1**). All treatments received standard agronomic management practices to be consistent with local conventional adzuki bean production.

**Table 1:** Target shade percentages for control (CK), red, blue, yellow, and green photo-selective nets.

Shade level	CK	RN	BN	YN	GN
Target Shade(%)	0.00	15	15	15	15
Actual Shade(%)	0.00	15.54	15.01	15.34	15.87

Photosynthetically active radiation (PAR) under each replica was monitored every 30 min, from 10:00 to 18:00, with values compared relative to CK to calculate the actual shading percentage. Measurements were conducted at the canopy apex.

### Spectral irradiance transmitted through photo-selective nets

**Figure 2:**
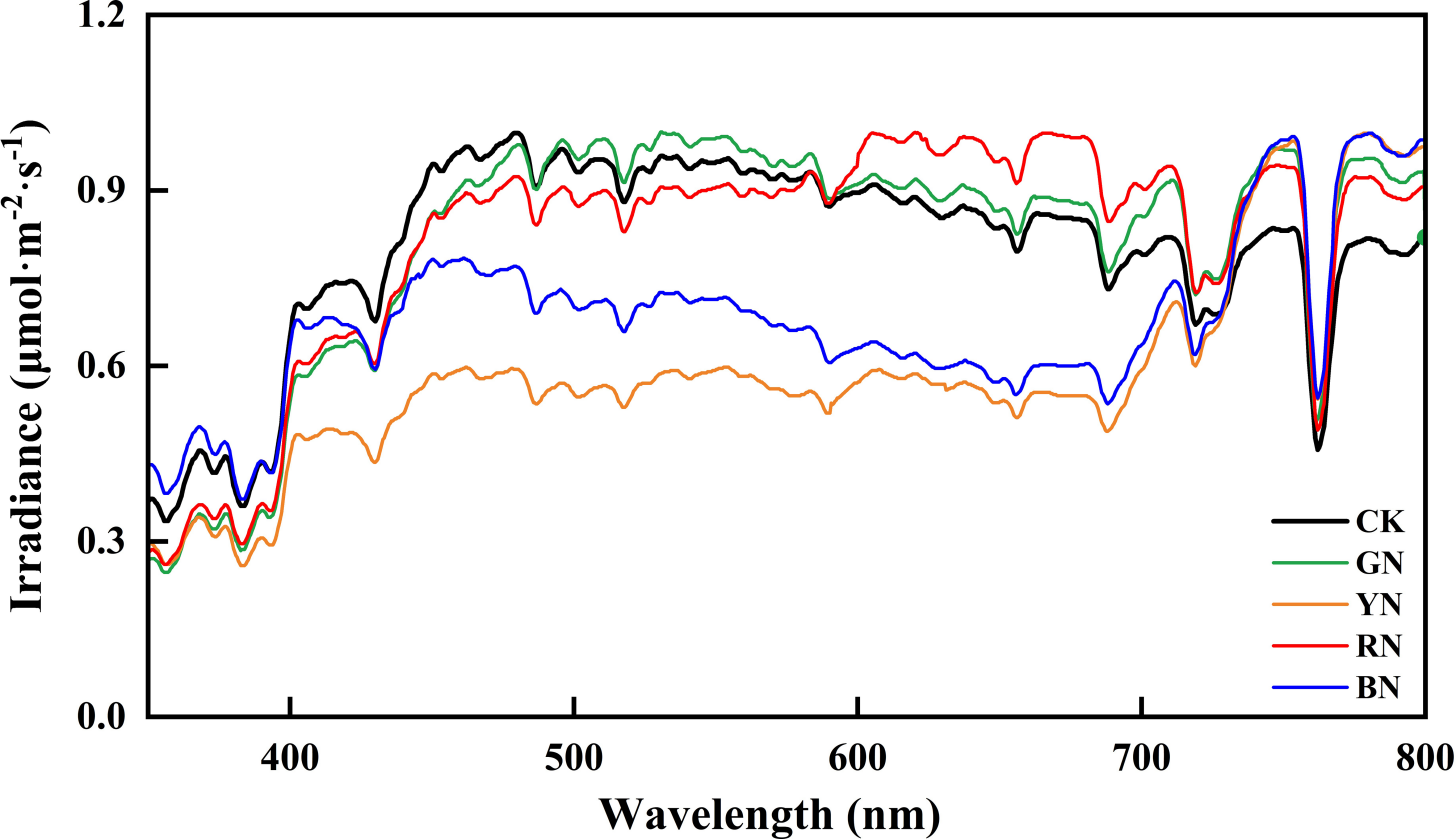
Spectral characteristics of different photo-selective net. CK, control; BN, blue photo-selective nets; RN, red photo-selective nets; YN, yellow photo-selective nets and GN, green photo-selective nets.

Under clear-sky conditions, spectral irradiance transmitted through each photo-selective nets were measured using a UniSpec-SC spectrometer (PP-Systems, Amesbury, UK), covering a wavelength range of 300–800 nm at 3.3 nm intervals. The spectral transmittance profiles for all treatments are presented in **Figure 2**.

### Growth parameter

Morphological parameters, including plant height, stem diameter, and leaf area, were measured at 7, 14, and 21 days after the treatments. Plant height was measured from the base of the lowest true leaf to apical meristem. Stem diameter was measured as the maximal thickness of main stem at the second internode. Leaf area (cm²) was calculated as length × maximum width × 0.71 (a cultivar-specific correction factor for *Vigna angularis*). Plant organs (roots, stems, and leaves) were separated, immediately placed in pre-labeled paper envelopes. They were then treated in an oven at 105°C for 30 minutes, then dried at 80°C until they reached a constant weight. After cooling at room temperature, the weight of them was obtained with a 0.01 g electronic balance and the dry mass calculated.

### Yield evaluation

At physiological maturity, fifteen representative plants were randomly sampled per treatment to evaluate yield components, including pod number per plant, grain number per pod, and 100-grain weight. The actual plot yield was calculated based on the components.

#### Determination of grain quality

##### Seed color

Objective color measurement of adzuki bean seeds was performed using a Minolta CR-400 chromameter (Minolta, Osaka, Japan), calibrated against standard white tile. The CIE L*, a*, b* color space was employed. L* represents lightness (0 = black, 100 = white). The a* value indicates the position on the red-green axis (+ = red, - = green), while b* indicates the position on yellow-blue axis (+ = yellow, - = blue) (Ibraheem et al., 2012).

##### Bean paste rate

Bean paste was extracted following the method reported by Sikora et al. (2018) with modifications: fresh beans (M, g) were soaked in distilled water (25 ± 1°C, 10 h), then steam-cooked at atmospheric pressure (1 h) using a laboratory boiler. The cooked adzuki beans were homogenized in a mortar using a pestle and sieved through a 60-mesh screen. The retention was collected and dried in a forced-air convection oven at 80 °C for 48 h until constant weight was achieved, yielding dry bean paste (W0, g). The bean paste yield (C, %) was calculated using the following formula:


C (%) = (W0/M)×100

##### Soluble sugars, soluble protein and starch

Total starch content was determined by enzymatic hydrolysis according to the Chinese National Standard GB 5009.9-2016; Soluble protein content was determined according to the Chinese National Standard GB 5009.6-2016. Soluble sugar content was determined by the anthrone colorimetric method as described by Li et al. (2022).

##### Total phenolic and total flavonoids content

Total phenolic content (TP) was quantified following the Folin-Ciocalteu method (Tian et al., 2016) using gallic acid as the standard compound. Results were expressed as mg gallic acid equivalents per gram dry weight (mg GAE g^-1^ DW). Total flavonoid content (TF) was measured using the aluminum chloride colorimetric assay (Sharma et al., 2018) with rutin as the standard. Absorbance was measured at 510 nm after 30 min incubation, and quantified against a rutin calibration curve. Results were expressed as mg rutin equivalents per gram dry weight (mg RE g^-1^ DW).

##### Element contents

A dried subsample (0.2 g) was ground to a fine powder and carefully digested with H_2_SO_4_-H_2_O_2_. Nitrogen (N) and phosphorus (P) contents were determined using a discrete automated analyzer (Smartchem 200, Alliance, France). Potassium (K) content in the resulting solution was measured using a flame photometer (ZA3000, ELE Instrument Co., Ltd., Stone, Staffordshire, Japan).

#### Photosynthetic physiological

##### Photosynthetic pigments

Chlorophyll (Chl) and carotenoid (Car) were extracted and quantified according to the ethanol extraction method described by Zhou et al. (2015). With this aim, fresh leaf tissue (0.1 g) was put into a test tube and added ten milliliters of 95% (v/v) ethanol, then the Chl and Car were extracted in the dark for 48 h, or until the tissue became bleached. The absorbance of the extract was then measured at 665 nm, 649 nm, and 470 nm using a microplate reader (Epoch2, BioTek Instruments, USA). The measured absorbance values at these wavelengths were used to calculate chlorophyll a (Chl a), chlorophyll b (Chl b), and carotenoid (Car) contents according to the following equations:


Chl a=(13.7×OD665−5.76×OD649)×0.03×1/m

Chl b=(25.8×OD649−7.6×OD665)×0.03×1/m

Car=(1000×OD470−2.05Ca−114.8Cb)/245

In the formula: Chl a, Chl b, Car-chlorophyll a, chlorophyll b, carotenoid content (mg·g^-1^ FW).

OD 649, OD 665, and OD 470 are the light absorption values of chlorophyll at wavelengths of 649 nm, 665 nm, and 470 nm, respectively.

##### Gas exchange parameters

At 7, 14, and 21 days post-treatment, net photosynthetic rate (Pn), stomatal conductance (Gs), intercellular CO_2_ concentration (Ci), and transpiration rate (Tr) of the third fully expanded leaf were assessed using a Li-6400 portable photosynthesis system (Li-COR, NE, USA) under 1200 μmol·m^-^²·s^-^¹ PAR, 400 μmol·mol^-^¹ CO_2_, and 25°C (Gao et al., 2020). Diurnal variations in photosynthetic parameters were measured at 21 days, with measurements taken every 2 hours from 7:00 to 17:00 on three plants, using the leaves at same developmental stage as samples (Tucci et al., 2010).

##### Chlorophyll fluorescence measurement

Fluorescence parameters, including initial fluorescence (Fo), maximum fluorescence (Fm), initial fluorescence (Fo’), and maximum fluorescence (Fm’), were measured using the MINI-PAM-II portable fluorescence analyzer (WALZ, Germany). The assessed parameters included: maximum photochemical efficiency (Fv/Fm), actual photochemical efficiency (ΦPSII) = (Fm’-Fs)/Fm’, photochemical quenching coefficient (qP) = (Fm’-Ft)/(Fm’-Fo’), and non-photochemical quenching coefficient (NPQ) = Fm/Fm’-1. The light intensity of 1200 μmol·m^-^²·s^-^¹ was used for the determination of NPQ and ΦPSII, as described by Ren et al. (2023b).

##### Anatomical structure of leaves

After 14 days of treatment, the paraffin sections of samples (5 mm × 5 mm) were fixed using a formalin-acetic acid-alcohol (FAA) fixative, dehydrated through an ethanol and xylene series, embedded in paraffin, and cross-sectioned to a thickness of 10 μm. The sections were then stained with safranin and fast green. Total leaf thickness, upper epidermis, palisade mesophyll, and spongy mesophyll thicknesses were measured using an optical microscope equipped with an eyepiece micrometer.

##### Antioxidant system

At 7, 14, and 21 days after treatment, the second fully - expanded trifoliate leaves were collected from Adzuki bean plants for quantification of antioxidant metabolites and key enzyme activities. The activities of peroxidase (POD), superoxide dismutase (SOD), and catalase (CAT), as well as the contents of malondialdehyde (MDA), hydrogen peroxide (H_2_O_2_), and superoxide anion radical (O_2_^-^), were assayed using commercial kits (Suzhou Geruisi Biotechnology Co., Ltd., Suzhou, China) following the manufacturer protocols.

### Data analysis

Data processing and statistical analyses were performed using Microsoft Excel 2020 (Microsoft Corp., USA) and DPS v14.10 (Hangzhou Ruifeng Information Technology Co., China). Significant differences between treatments were determined by Duncan’s multiple range test at P ≤ 0.05. Graphical presentations were generated with Origin Pro 2024 (Origin Lab Corp., USA).

## Results

### Spectral properties and par transmittance of photo-selective nets

Compared to CK, the red (RN), blue (BN), yellow (YN), and green nets (GN) provided shading intensities of 15.54%, 15.01%, 15.34%, and 15.87%, respectively, as shown by PAR transmittance reduction (**Table 1**). Distinct spectral transmission profiles were observed among photo-selective nets (**Figure 2**). BN exhibited significantly higher transmittance in blue (B, 420–460 nm) and green (500–570 nm) wavebands compared to other nets. RN significantly enhanced transmittance in red (640–680 nm) and far-red (690–750 nm) regions. YN reduced ultraviolet (280–400 nm) and blue (420–460 nm) transmittance, but increased yellow (570–600 nm), red (640–680 nm), and far-red (690–750 nm) transmission. GN showed significantly elevated transmittance in green (500–570 nm) and yellow (570–600 nm) regions relative to other treatments.

### Effects of photo-selective nets on the growth traits of adzuki bean

Varying light qualities and intensities exert profound impacts on the growth performance of adzuki beans across distinct growth stages. Plant growth parameters of adzuki bean under photo-selective nets are shown in **Table 2**. At 7 d - 21 d after treatment, the plant height of RN treatment was significantly higher than other treatments, increased by 8.23%, 14.24% and 12.23% relative to CK, respectively. The BN treatment enhanced stem thickness by 3.90%, 3.56%, and 8.15% compared with CK, respectively, and all light-selective net treatments significantly increased the leaf area of adzuki beans. At 7 d after treatment, the leaf area of BN treatment was significantly higher than other treatments, and at 14 d - 21 d, YN treatment was significantly higher than other treatments and reached the highest value, with increases of 20.47% and 27.70%, respectively, compared with control. Different photo-selective net treatments exerted more pronounced effects on the dry weight of adzuki bean. The BN treatment was significantly higher than other treatments at 7–21 d in root dry weight and root-shoot ratio, with root dry weight higher than CK by 71.43%, 5.00%, and 21.62%, respectively; root-shoot ratio was higher than CK by 53.85%, 5.56%, and 23.53%, respectively. The RN treatment was significantly higher than other treatments at 7 d and 14 d in stem dry weight, leaf dry weight, and total dry weight, respectively, increasing 61.54%, 33.33%, 40.68% and 18.42%, 24.64% and 16.54% over CK, respectively. At 21 d after treatment, YN significantly increased stem dry weight, leaf dry weight and total dry weight by 47.69%, 63.82% and 48.63%, respectively, compared with CK, followed by RN treatment which significantly increased stem dry weight, leaf dry weight and total dry weight by 20%, 28.30% and 18.04% compared with CK, respectively. In summary, different light selective networks promoted the growth of adzuki bean in different degrees. RN was shown to significantly plant height, leaf area, above ground dry weight and total dry weight whereas BN significantly promoted stem diameter, root dry weight and root-shoot ratio. Collectively, BN and RN differentially improved adzuki bean growth via distinct morphological adaptations.

**Table 2:** Influence of photo-selective nets on growth of adzuki bean.

Processing time	Treatment	Plant height (cm)	Stem diameter (mm)	Leaf area (cm^2^)	Root DW (g plant^-1^)	Stem DW (g plant^-1^)	Leaf DW (g plant^-1^)	Root-shoot ratio	Total DW (g plant^-1^)
7d	CK	18.28 ± 0.62bc	3.08 ± 0.08ab	30.40 ± 1.13c	0.07 ± 0.007c	0.13 ± 0.003b	0.39 ± 0.015cd	0.13 ± 0.015c	0.59 ± 0.017c
	RN	19.92 ± 0.47a	3.06 ± 0.05ab	37.79 ± 0.63a	0.10 ± 0.001b	0.21 ± 0.026a	0.52 ± 0.010a	0.14 ± 0.005bc	0.83 ± 0.029a
	BN	17.86 ± 0.50c	3.20 ± 0.07a	32.18 ± 1.81bc	0.12 ± 0.014a	0.14 ± 0.008b	0.47 ± 0.006b	0.20 ± 0.025a	0.73 ± 0.008b
	YN	18.84 ± 0.68b	2.99 ± 0.18b	33.65 ± 2.19b	0.08 ± 0.004c	0.13 ± 0.004b	0.42 ± 0.045bc	0.14 ± 0.020bc	0.63 ± 0.045c
	GN	17.74 ± 0.45c	2.75 ± 0.13c	31.22 ± 0.99c	0.08 ± 0.008c	0.14 ± 0.005b	0.37 ± 0.040d	0.16 ± 0.013b	0.58 ± 0.048c
14d	CK	21.06 ± 0.57c	3.37 ± 0.29ab	44.31 ± 1.11d	0.20 ± 0.006b	0.38 ± 0.009b	0.69 ± 0.046cd	0.18 ± 0.002a	1.27 ± 0.043bc
	RN	24.06 ± 0.44a	3.32 ± 0.09ab	46.64 ± 0.45c	0.17 ± 0.011c	0.45 ± 0.015a	0.86 ± 0.027a	0.13 ± 0.010b	1.48 ± 0.019a
	BN	23.04 ± 0.42b	3.49 ± 0.05a	46.39 ± 1.23c	0.21 ± 0.010a	0.37 ± 0.019b	0.75 ± 0.054bc	0.19 ± 0.009a	1.33 ± 0.071b
	YN	23.08 ± 0.43b	3.32 ± 0.10ab	53.38 ± 0.61a	0.11 ± 0.004d	0.38 ± 0.010b	0.77 ± 0.001b	0.09 ± 0.004c	1.25 ± 0.010c
	GN	23.38 ± 0.39b	3.25 ± 0.11b	50.51 ± 1.23b	0.12 ± 0.004d	0.27 ± 0.006c	0.64 ± 0.030d	0.13 ± 0.003b	1.02 ± 0.027d
21d	CK	28.60 ± 0.89b	4.66 ± 0.07c	46.79 ± 0.82e	0.37 ± 0.027b	0.65 ± 0.047c	1.52 ± 0.058c	0.17 ± 0.014b	2.55 ± 0.179c
	RN	32.10 ± 0.82a	4.66 ± 0.29c	51.97 ± 0.73c	0.28 ± 0.006c	0.78 ± 0.062b	1.95 ± 0.031b	0.10 ± 0.001d	3.01 ± 0.044b
	BN	29.37 ± 0.37b	5.04 ± 0.07a	50.14 ± 1.08d	0.45 ± 0.038a	0.78 ± 0.029b	1.31 ± 0.045d	0.21 ± 0.017a	2.54 ± 0.144c
	YN	28.80 ± 0.27b	4.66 ± 0.05c	59.75 ± 1.89a	0.34 ± 0.012b	0.96 ± 0.036a	2.49 ± 0.170a	0.10 ± 0.007d	3.79 ± 0.130a
	GN	31.60 ± 1.88a	4.75 ± 0.08b	57.66 ± 1.91b	0.27 ± 0.006c	0.59 ± 0.026c	1.58 ± 0.038c	0.13 ± 0.006c	2.44 ± 0.049c

Values are means ± SD. 7 d, 14 d, and 21 d indicate the 7 th, 14 th, and 21st day of processing, respectively. Different letters (a - e) in the same column denote significant differences among treatments at P ≤ 0.05 as determined by Duncan’s multiple range test. Values represent mean ± SD (n = 3). DW, dry weight; CK, control; RN, red photo-selective nets; BN, blue; YN, yellow and GN, green.

### Photo-selective net effects on photosynthetic pigment in adzuki bean

**Figure 3:**
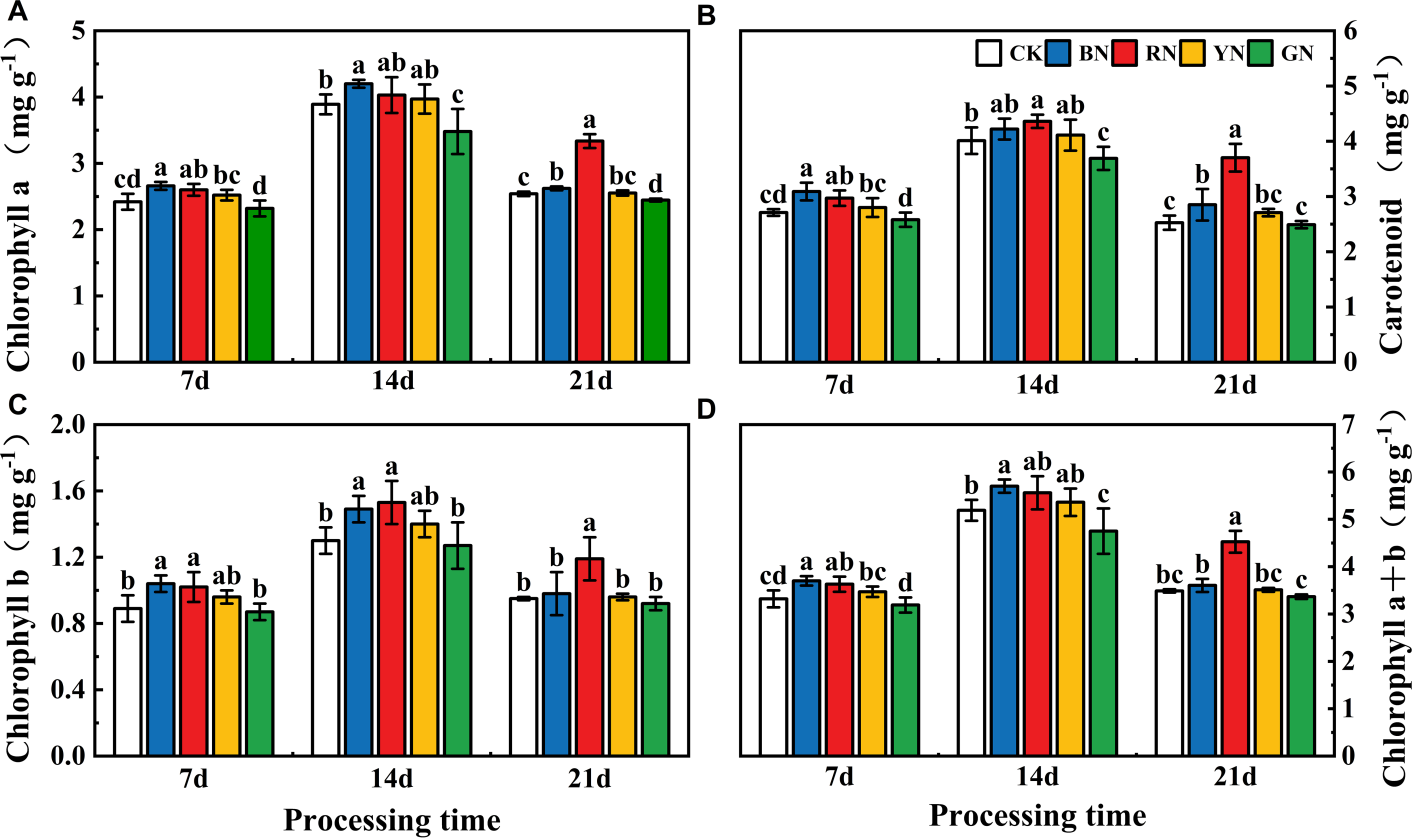
Effects of photo-selective nets on photosynthetic pigment contents in adzuki bean leaves at different treatment times. **(A)** Chlorophyll a content, **(B)** Carotenoid content, **(C)** Chlorophyll b content, **(D)** Chlorophyll a+b content. Values represent means ± SD. Error bars denote standard deviations. Different letters (a-e) in the same column indicate significant differences among treatments at P ≤ 0.05 via Duncan’s multiple range test.

RN and BN significantly increased chlorophyll and carotenoid contents compared with CK throughout the treatment periods, while GN caused a reduction in photosynthetic pigments (**Figure 3**). BN and RN significantly increased chl a content by 3.23% - 9.92% and 3.60% - 31.34% relative to CK, while GN induced a decrease of 3.70% - 10.54% (P < 0.05; **Figure 3**). BN and RN significantly enhanced chl b content by 3.16% - 16.85% and 14.61% - 25.26% compared with CK, whereas GN led to a 2.30% - 3.26% reduction (P < 0.05; **Figure 3**). Compared with control, the carotenoid content of BN and RN treatment plants was increased by 4.49% - 14.02% and 1.43% - 9.59%, respectively, while that of GN treatment plants was decreased by 1.43%-7.98%. Compared with CK plants, the chl a + b contents of plants treated with BN and RN increased by 3.33% - 11.45% and 0.63% - 9.33% respectively, while those treated with GN decreased by 3.50% - 8.48% (**Figure 3**). Photosynthetic pigment contents (chlorophyll and carotenoids) in adzuki bean leaves increased initially then declined during the experimental period. Peak photosynthetic pigment concentrations occurred at 14 days after treatment. Thus, both RN and BN treatments increased the photosynthetic pigment contents of adzuki bean leaves. The content of carotenoids was consistent with the variation trend of chlorophyll content (**Figure 3**).

### Effects of photo-selective nets on photosynthetic gas exchange in adzuki bean

**Figure 4:**
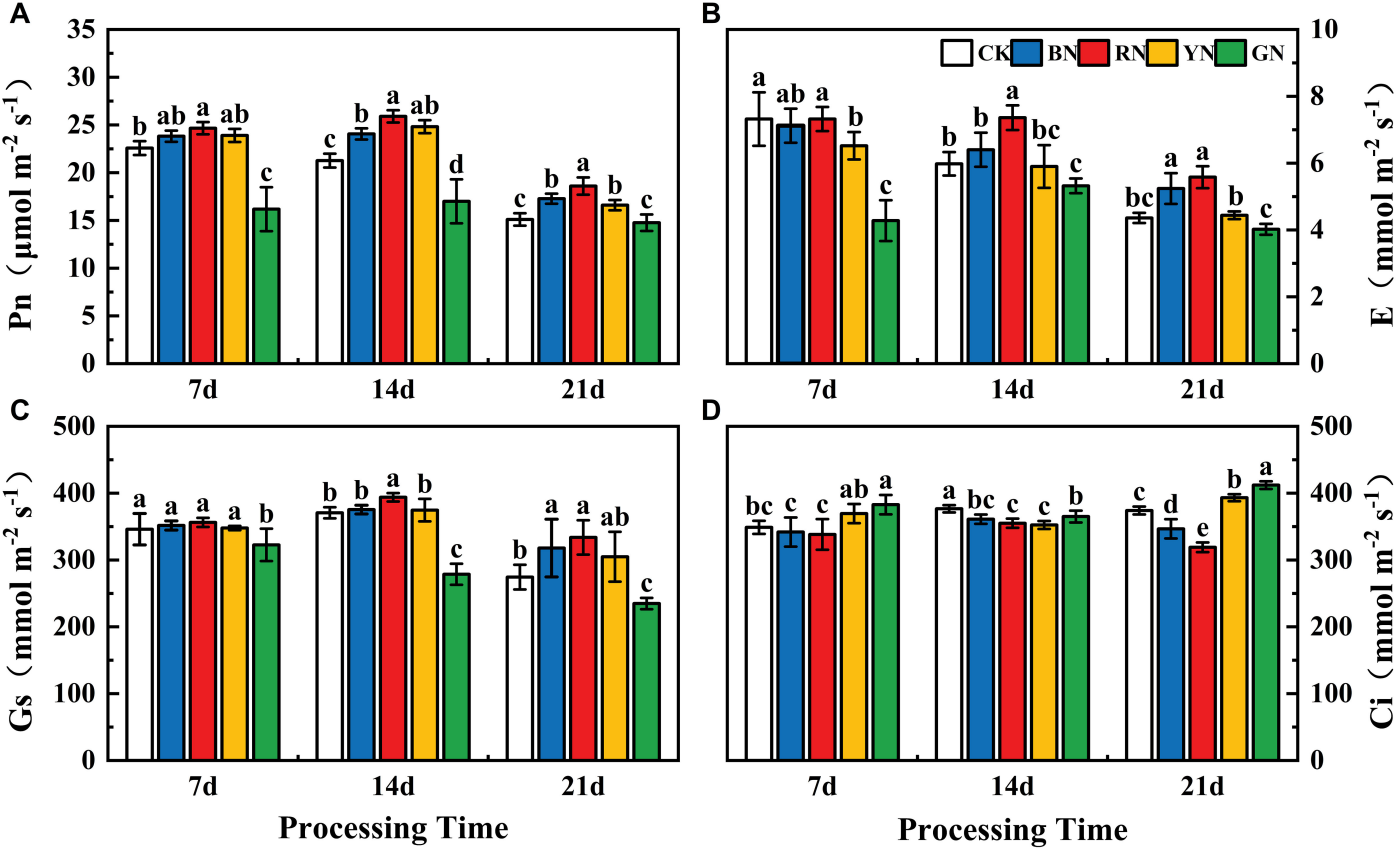
Effects of photo-selective nets on photosynthetic parameters in adzuki bean leaves at different processing times. **(A)** Net photosynthetic rate (Pn), **(B)** transpiration rate **(E)**, **(C)** stomatal conductance (Gs), **(D)** intercellular CO_2_ concentration (Ci).

Photo-selective shade nets effectively influenced the gas exchange parameters of adzuki bean leaves. Pn, Tr and Gs exhibited a pattern of initial increase followed by a decrease as the treatment duration progressed. At 7 d after treatment, Pn, Tr and Gs did not differ significantly among the treatments, with the highest values observed under RN treatment, followed by BN and YN. At 14 d after treatment, the highest level was observed, where Pn, Gs, and Tr in the RN treatment were significantly higher than other treatments, showing to be increased by 21.83%, 6.26%, and 23.08% relative to CK, respectively (**Figures 4A–C**). At 21 d after treatment, Pn, Gs, and Tr in RN and BN treatments were significantly higher than CK, increasing by 23.18%, 15.82%, 27.98% and 14.44%, 21.65%, 20.18%, respectively, whereas those in GN treated plants decreased by 2.25%, 14.43%, and 7.80% relative to CK, respectively (**Figures 4A–C**). The trend of Ci was opposite to those of Pn, Tr, and Gs. It reached the peak under YN treatment and the lowest level under RN treatment, indicating that the opening of stomata induced by RN more effectively that promotes CO_2_ assimilation, thereby increasing the Pn (**Figure 4**).

**Figure 5:**
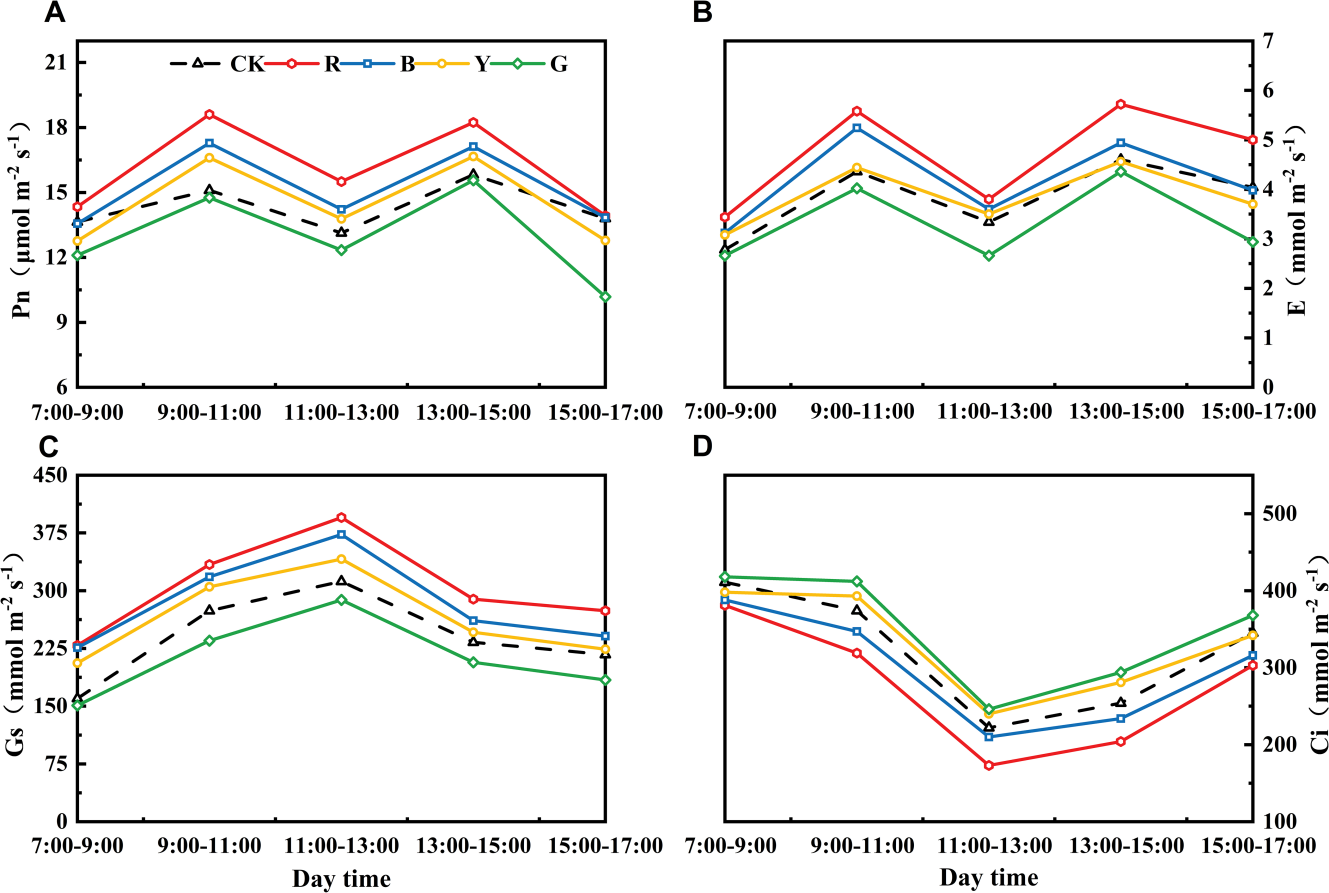
Effects of photo-selective nets on diurnal variation in photosynthetic parameters of adzuki bean leaves after 21 days of treatment. **(A)** Net photosynthetic rate (Pn). **(B)** Transpiration rate (E). **(C)** Stomatal conductance (Gs). **(D)** Intercellular CO2 concentration (Ci).

By analyzing and treating the diurnal variation of photosynthesis in adzuki beans for 21 days (**Figure 5**), the differences among different light selection networks under different treatment times were consistent. Among them, the Pn, Tr and Gs values of the plants treated with RN were the highest. Over a diurnal cycle, the diurnal variation of Pn and Tr displayed an “M”-shaped curve, with maxima at 9:00 - 11:00 and 13:00 - 15:00, and a minimum between 11:00 - 13:00. The “midday photosynthetic depression” (Tucci et al., 2010) in control (CK) and green net (GN) plants was significantly more pronounced during 11:00 - 13:00 than in other treatments (**Figure 5**). Compared with that in CK, the Pn of BN, RN, and YN treatments increased by 18.05%, 8.30%, and 4.95%, respectively. Consequently, photo-selective nets enhanced leaf stress tolerance, increased net photosynthetic rate (Pn), and alleviated midday depression in adzuki bean. Stomatal conductance (Gs) diurnal patterns showed unimodal curves across the treatments. The diurnal variation in Gs showed a trend of initial increase followed by decrease, peaking at 11:00 - 13:00 before declining (**Figure 5**). Intercellular CO_2_ concentration (Ci) dynamics peaked in the morning, declined to a midday minimum, and partially recovered in the afternoon, which exhibited an inverse relationship with net photosynthetic rate (Pn) (**Figure 5**).

### Effects of photo-selective nets on chlorophyll fluorescence in adzuki bean

**Figure 6:**
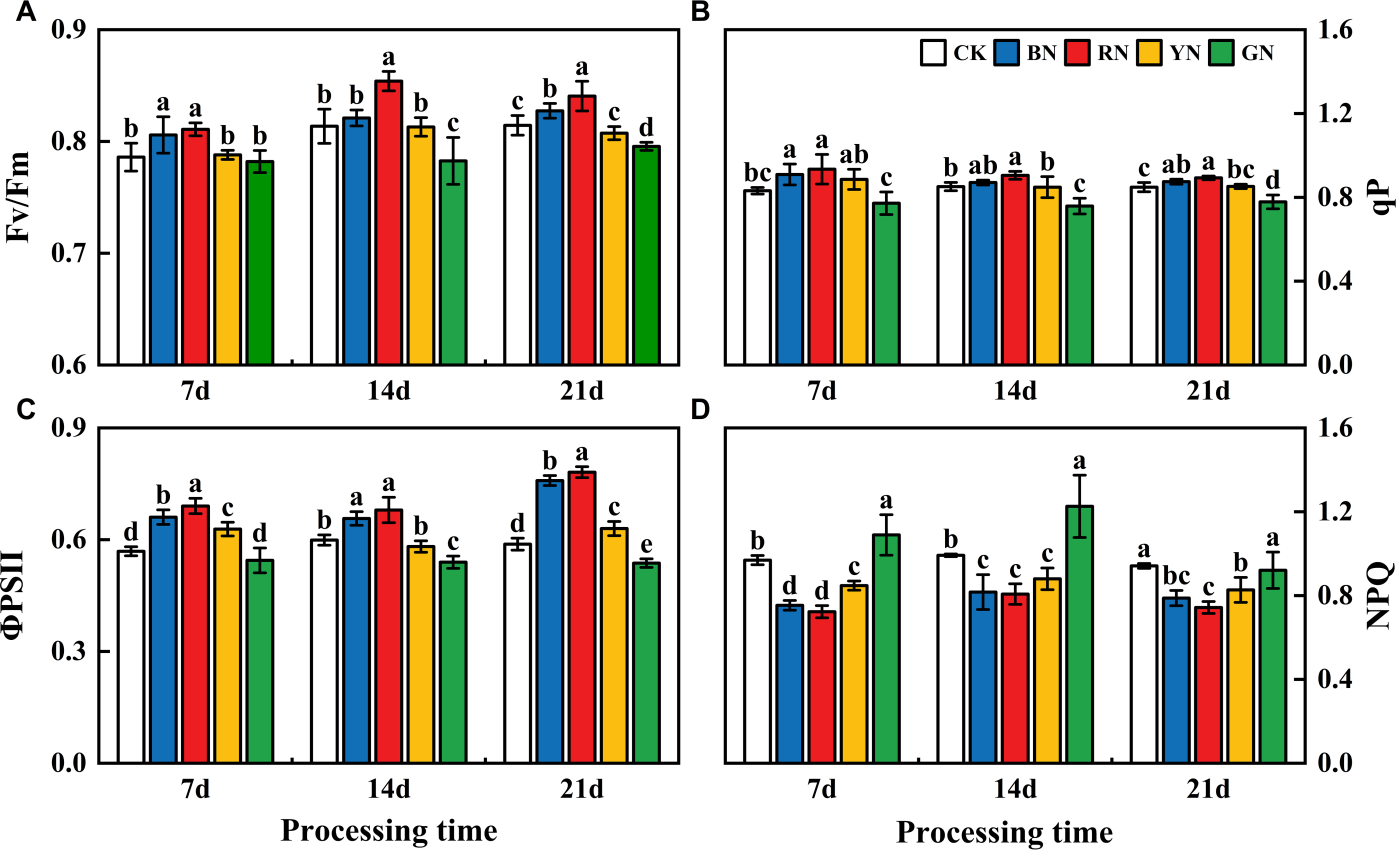
Effects of photo-selective nets on fluorescence parameters of adzuki bean leaves at different processing times. **(A)** Maximum photochemical efficiency of PSII under dark adaptation (Fv/Fm), **(B)** Photochemical quenching coefficient (qP), **(C)** Actual photochemical efficiency (ΦPSII), **(D)** Non-photochemical quenching coefficient (NPQ).

Photo-selective nets exerted significant influences on the chlorophyll fluorescence parameters of adzuki bean leaves. The chlorophyll fluorescence parameters (Fv/Fm, qP, ΦPSII) of each treatment displayed a descending order as follows: RN > BN >YN > CK> GN. In treatment 7 d - 21 d, compared with that in CK plants, Fv/Fm in RN and BN treatments increased by 3.14% - 5.04% and 0.91 - 2.52% relative to CK, whereas in GN decreased by 0.51 - 3.81% (**Figure 6A**). In treatment 7 d - 21 d, ΦPSII in RN and BN treatments increased by 6.31% - 32.85% and 9.65% - 29.01% relative to CK, while GN treatment caused a reduction of 4.31% - 9.92%. In treatment 7 d - 21 d, qP in RN and BN treatments increased by 5.20% - 12.63% and 2.26% - 9.33%, whereas GN treatment led to a 6.94% - 8.30% decrease (**Figure 6B**). Therefore, BN and RN significantly enhanced Fv/Fm and ΦPSII under partially closed reaction centers, whereas GN enhanced NPQ in adzuki bean plants.

### Effects of photo-selective nets on adzuki bean leaf anatomical structure

**Figure 7:**
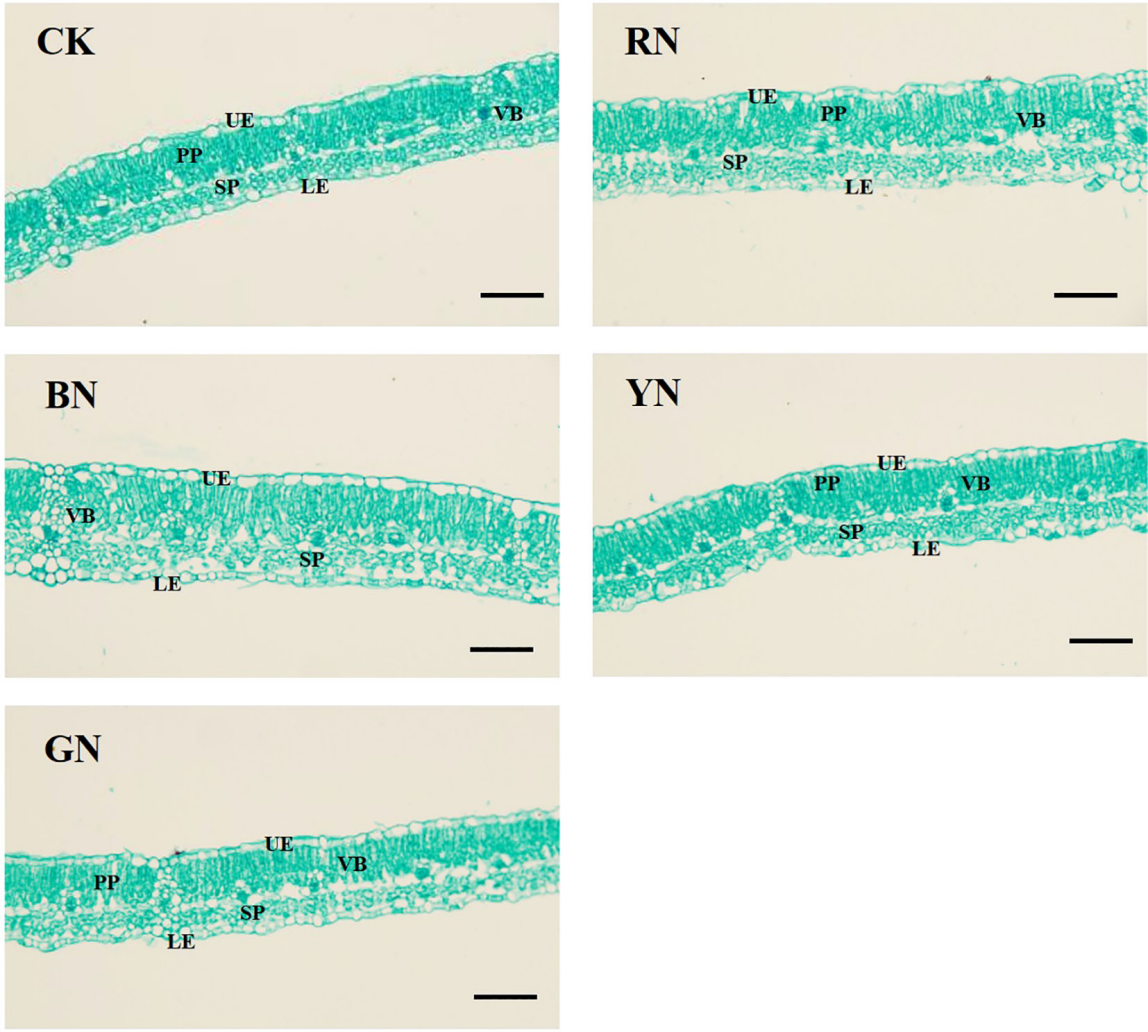
Leaf anatomical structure of adzuki bean under different photo-selective nets. UE, upper epidermis; PP, palisade parenchyma; SP, spongy parenchyma; LE, lower epidermis; VB, vascular bundle. Scale bars = 50 μm.

Anatomical structures of adzuki bean leaves were significantly influenced by different photo-selective nets (**Figure 7**; **Table 3**). Under BN and RN photo-selective nets, leaf sections exhibited palisade tissues with uniform cell size and tight arrangement. BN and RN photo-selective nets promoted larger palisade cells and additional layers of spongy mesophyll in leaf sections (**Figures 7A**). Furthermore, BN treatment increased the maximum values of total leaf thickness (TLT), palisade parenchyma (PP), spongy parenchyma (SP), and upper epidermis (UP), all showing to be statistically significant increases relative to CK and other photo-selective nets (**Figure 7**; **Table 3**). Specifically, leaf thickness under BN, RN, and GN treatments increased by 27.96%, 12.15%, and 7.96% relative to CK, respectively. The palisade and spongy tissues in RN and BN treatments increased by 8.68% and 49.74% (palisade), and 21.01% and 66.96% (spongy) relative to CK, respectively (**Table 3**). These morphological adaptations indicate that enhanced spatial organization of leaf tissues for more efficient light energy absorption potentially improves photosynthetic performance. However, no significant differences were observed in lower epidermal thickness or cellular arrangement across treatments.

**Table 3:** Anatomical structure of adzuki beans leaves under different photo-selective net treatments.

Treatment	Palisade parenchyma (μm)	Spongy parenchyma (μm)	Upper epidermis (μm)	Lower epidermis (μm)	Total thickness (μm)	Ratio of palisade to spongy tissue
CK	34.45 ± 2.88c	15.68 ± 2.17e	8.53 ± 1.28ab	7.29 ± 2.05a	67.24 ± 1.74c	2.25 ± 0.44a
RN	37.44 ± 2.10b	23.48 ± 2.35b	7.78 ± 1.42b	6.63 ± 1.60a	75.41 ± 4.17b	1.61 ± 0.17b
BN	41.69 ± 3.72a	26.18 ± 3.22a	9.47 ± 0.87a	7.97 ± 1.24a	86.04 ± 4.57a	1.62 ± 0.27b
YN	33.64 ± 1.92c	18.39 ± 1.67d	6.57 ± 0.81c	7.37 ± 1.38a	67.61 ± 1.81c	1.84 ± 0.13b
GN	34.40 ± 2.08c	20.53 ± 1.90c	9.12 ± 1.50a	7.58 ± 1.65a	72.59 ± 1.91b	1.69 ± 0.19b

Different letters (a - d) in the same column denote significant differences among treatments at P ≤ 0.05 via Duncan’s multiple range test. Values represent mean ± SD (n = 3).

### Effects of photo-selective nets on adzuki bean antioxidant system

**Figure 8:**
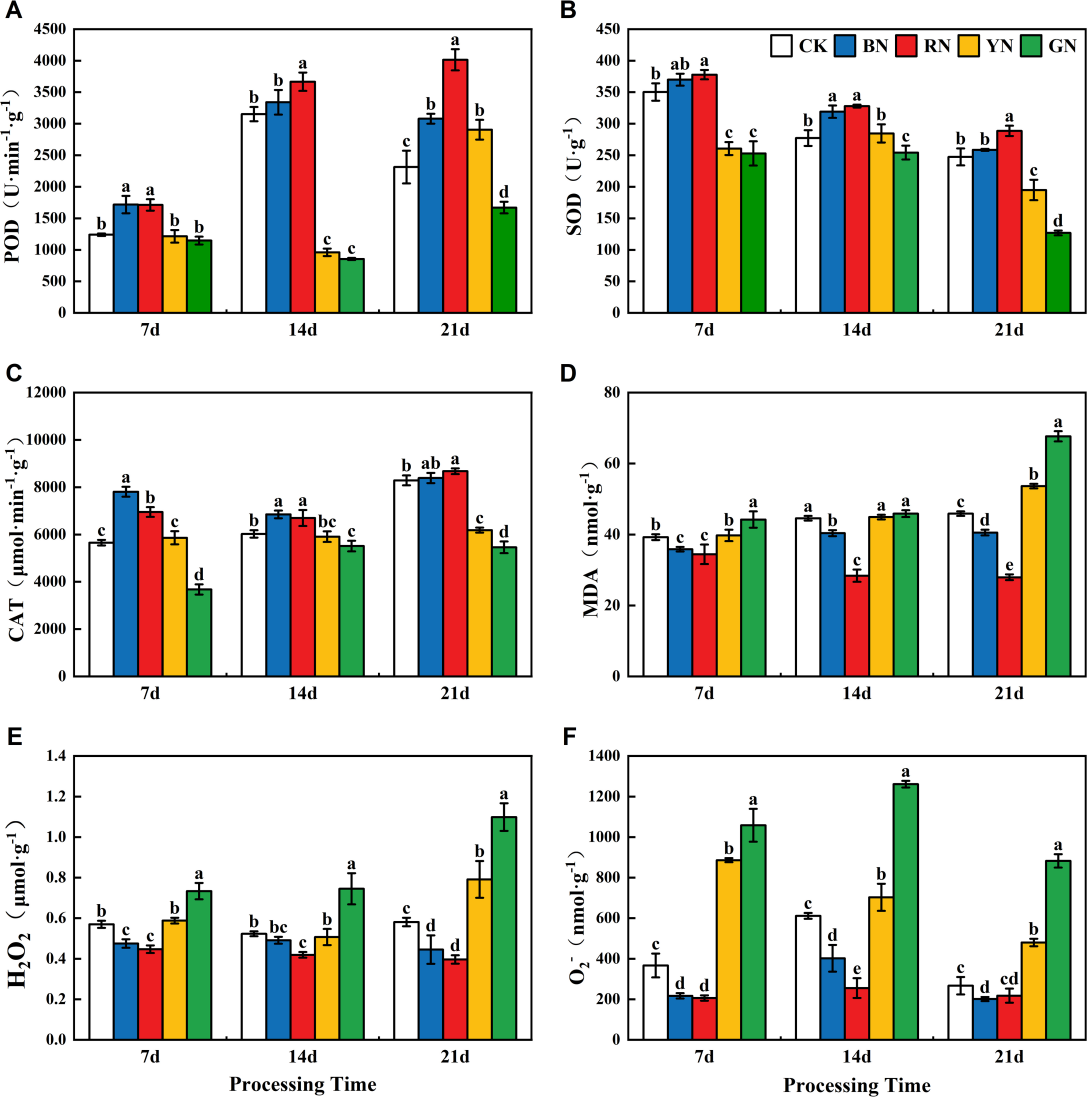
Effects of photo-selective nets on reactive oxygen species, malondialdehyde (MDA) content, and antioxidant enzyme activities in adzuki bean leaves across treatment durations. **(A)** POD activity, **(B)** SOD activity, **(C)** CAT activity, **(D)** MDA content, **(E)** H_2_O_2_, **(F)** O_2_^-^ (superoxide anion).

Reactive oxygen species (ROS) accumulation in adzuki bean leaves exhibited significant variation across photo-selective net treatments (**Figure 8**). The ROS content under different treatments exhibited following descending order: GN > YN> CK > BN > RN. During the treatment, the contents of H_2_O_2_ and MDA exhibited an increasing trend, whereas the O_2_^-^ content initially increased and then decreased. The ROS levels in YN and GN treatments were significantly higher than those in CK (**Figure 8E**), while RN and BN treatments showed significantly lower ROS levels than CK. The trend of malondialdehyde (MDA) content under different photo-selective nets was consistent with the levels of ROS (**Figure 8**). At 21d, GN, the MDA content in GN and YN treated leaves was 47.54% and 16.90% higher than CK, respectively, BN and RN treated leaves showed 11.62% and 39.08% lower MDA content than CK, respectively. Superoxide dismutase (SOD) serves as the primary defense in plant antioxidant system, which effectively scavenges excessive superoxide radicals (O_2_^-^) to maintain intracellular redox balance. In the present study, the activities of POD, SOD and CAT in RN and BN treated plants were significantly higher than those in CK, whereas GN and YN treated leaves showed lower activities (**Figures 8A–C**). Peroxidase (POD) and catalase (CAT) are crucial redox enzymes that scavenge ROS and play crucial roles in plant physiology. POD activity in adzuki bean leaves increased throughout the experiment, peaking at 21 days under photo-selective nets. At this time, POD activity in RN, BN, and YN treatments was 73.52%, 33.11%, and 25.58% higher than CK, respectively, while GN treatment showed a 27.77% decrease. CAT activity in leaves exhibited a trend of initial decrease followed by increase, peaking at 21 days. RN and BN treatments enhanced CAT activity by 4.65% and 1.84% relative to CK, whereas GN and YN treatments decreased by 34.16% and 25.40% decreases, respectively. In conclusion, BN and RN treatments enhanced the SOD, POD, and CAT activities in adzuki bean leaves and reduced MDA content and consequently decreased ROS accumulation.

### Effects of photo-selective nets on adzuki bean grain quality parameters

The appearance and nutritional qualities of adzuki bean kernels were evaluated under different photo-selective net treatments. The results showed that RN, BN and YN significantly increased the L* value of adzuki bean seed color, while RN and BN enhanced the a* value and BN significantly increased the value of b*. Overall, RN and BN treatments improved seed color and enhanced the appearance quality of adzuki beans. Quality evaluation showed no significant difference in bean paste rate among the treatments. However, significant differences were observed in starch, soluble protein, and soluble sugar contents under different photo-selective nets. The RN treatment increased soluble sugar and starch contents by 4.44% and 4.69%, respectively, compared to CK. The BN treatment promoted soluble protein and amino acid contents by 7.69% and 9.55%, respectively, relative to CK (**Table 4**). Total phenolics and flavonoids in adzuki bean seeds varied significantly under different photo-selective net treatments. Compared to control, all treatments significantly increased the contents of total phenolics and flavonoids. The highest total phenolics were observed in RN-treated seeds, followed by GN > YN > BN > CK, with increases of 20.00%, 13.75%, 11.25% and 1.88%, respectively. The total flavonoid content peaked in GN treated seeds, followed by BN>RN>YN>CK, showing to be increased by 51.21%, 38.05%, 31.22%, and 11.71%, respectively (**Table 4**). These findings indicate that photo-selective net treatments enhance the contents of total phenolics and flavonoids in adzuki bean grains.

**Table 4:** Effects of photo-selective nets on grain quality of adzuki bean.

Treatment	Bean Paste Rate (%)	Soluble sugars content (%)	Soluble proteins content (%)	Starch content (%)	amino acid content (mg/g)	L*	a*	b*	Total phenolic content (mg g^-1^)	Total flavonoid content (mg g^-1^)
CK	20.24 ± 0.49ab	3.38 ± 0.16ab	12.49 ± 1.92ab	54.84 ± 0.43b	1.78 ± 0.01b	25.29 ± 1.92ab	12.98 ± 0.91b	6.37 ± 1.36a	1.60 ± 0.06c	2.05 ± 0.06d
RN	20.70 ± 0.15a	3.53 ± 0.06a	11.11 ± 0.96b	57.41 ± 0.35a	1.69 ± 0.01c	26.63 ± 2.65a	14.47 ± 2.51a	6.27 ± 1.31ab	1.92 ± 0.04a	2.69 ± 0.05b
BN	20.35 ± 0.54ab	3.51 ± 0.06a	13.45 ± 0.20a	53.90 ± 0.56b	1.95 ± 0.03a	26.26 ± 1.50a	14.43 ± 0.95a	6.64 ± 0.94a	1.63 ± 0.05c	2.83 ± 0.10b
YN	18.95 ± 0.74c	3.37 ± 0.05ab	11.91 ± 0.64ab	48.56 ± 1.31c	1.60 ± 0.02d	26.55 ± 2.44a	12.87 ± 0.81b	5.57 ± 0.73b	1.78 ± 0.04b	2.29 ± 0.10c
GN	19.36 ± 0.87bc	3.30 ± 0.10b	12.07 ± 0.35ab	46.45 ± 2.69c	1.58 ± 0.03d	24.28 ± 1.94b	13.78 ± 1.58ab	6.15 ± 1.02ab	1.82 ± 0.03b	3.10 ± 0.09a

Different letters (a - d) in the same column denote significant differences among treatments at P ≤ 0.05 via Duncan’s multiple range test. Values represent mean ± SD.

### Photo-selective nets effects on the macronutrient contents of adzuki bean

**Figure 9:**
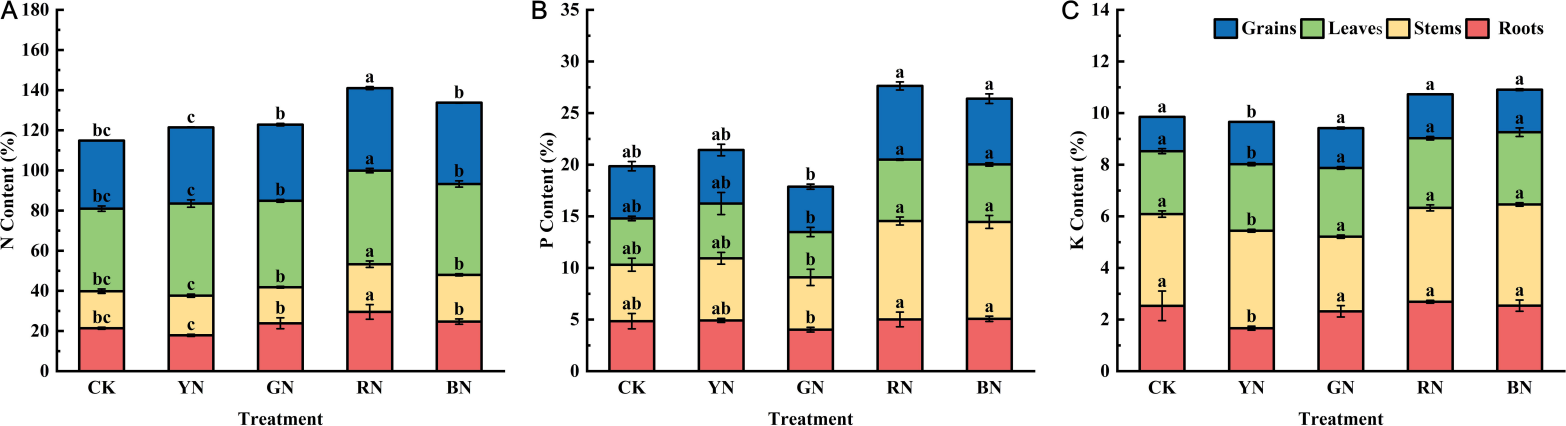
Effects of photo-selective nets on element contents in roots, stems, leaves, and grains of adzuki bean at harvest stage. **(A)** N content, **(B)** P content, **(C)** K content.

As primary macronutrients, nitrogen (N), phosphorus (P), and potassium (K) are fundamental to plant growth and development. During the harvest period, the concentrations of N, P and K in organs of adzuki beans (roots, stems, leaves and grains) were determined. Significant differences in plant nutrient balance were observed across photo-selective net treatments. Of which, RN treatment induced the highest nitrogen content in adzuki bean tissues, with leaves exhibiting peak accumulation (46.65% dry weight), followed by grain (41.09%), roots (29.52%), and stems (23.76%) (**Figure 9**). Under CK treatment, the nitrogen content in the leaves and grains of adzuki beans was at its lowest level, indicating that different photo-selective nets can enhanced the translocation of nitrogen to both the grains and leaves. Phosphorus concentration in roots, stems, leaves and grain of adzuki bean were significantly influenced by the photo-selective net treatments. Among them, RN and BN significantly increased phosphorus content in all tested tissues, whereas the total phosphorus content was lowest under GN treatment. As shown in **Figure 9**, potassium concentration of adzuki bean was also significantly influenced by the various photo-selective net treatments. Compared to control, all photo-selective net treatments significantly the increased potassium content in stems, leaves, and grains of adzuki beans.

### Effects of photo-selective nets on the yield of adzuki bean

As shown in **Table 5**, the hundred-grain weight, pod number per plant, and seed number per pod of adzuki beans under different photo-selective net treatments were counted, and the yield per hectare was calculated. Significant differences were observed in the yield, hundred weights, Pod number per plant of adzuki bean were varied across the treatments. Compared with CK, the yield of RN and BN treated plants increased by 15.05% and 10.63%, respectively, whereas GN treatment decreased yield by 8.56%. The pod number per plant of plants in RN and BN treatments increased by 8.45% and 7.18% relative to CK, whereas GN treatment showed a 4.44% decrease. The hundred-grain weight in RN and BN treatments increased by 3.82% and 2.99% compared with CK, respectively, while GN treatment caused a 4.60% reduction. RN and BN treatments significantly increased grain length and width by 5.07%, 7.99% and 4.79%, 3.19% relative to CK, respectively, whereas GN treatment led to 0.56% and 2.43% decreases. Seeds per pod did not differ significantly among treatments.

**Table 5:** Effect of photo-selective nets on the yield of adzuki bean.

Treatment	Grain length (mm)	Grain width (mm)	Hundred-grain weight (g)	Pod number per plant(number)	Seed number per pod (number)	Yield (kg ha^-1^)
CK	7.10 ± 0.50bc	5.76 ± 0.22bc	16.74 ± 0.26bc	39.42 ± 7.76bc	6.80 ± 0.68a	2895.10 ± 10.84b
RN	7.46 ± 0.39a	6.22 ± 0.26a	17.38 ± 0.27a	42.75 ± 1.06a	7.00 ± 1.00a	3330.80 ± 52.24a
BN	7.44 ± 0.61ab	5.95 ± 0.22ab	17.24 ± 0.26ab	42.25 ± 1.42ab	6.93 ± 0.70a	3202.88 ± 42.12a
YN	7.10 ± 0.34bc	5.80 ± 0.69bc	16.45 ± 0.36cd	39.92 ± 4.08abc	6.80 ± 0.77a	2900.32 ± 31.83b
GN	7.06 ± 0.46c	5.62 ± 0.25c	15.97 ± 0.26d	37.67 ± 1.97c	6.80 ± 0.68a	2647.39 ± 20.42c

Different letters (a - d) in the same column denote significant differences among treatments at P ≤ 0.05 via Duncan’s multiple range test. Values represent mean ± SD.

### Cluster heatmap analysis

**Figure 10:**
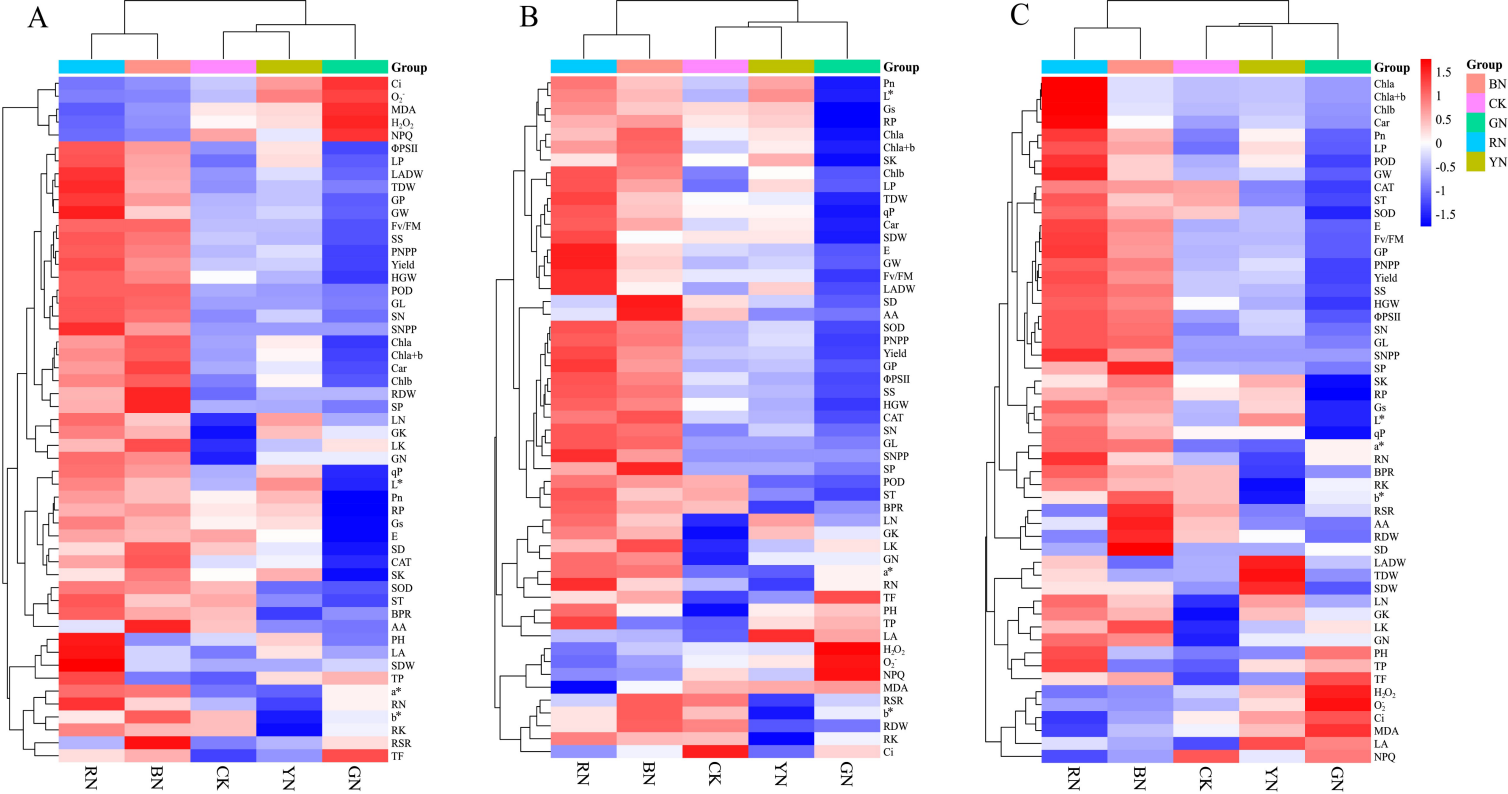
Cluster heat map analysis results of the phenotypic traits, photosynthetic indicators, antioxidant capacity, nutritional components, yield and quality of adzuki beans by photo-selective nets. **(A)** 7 days, **(B)** 14 days, **(C)** 21 days. Abbreviations of traits are listed in the abbreviations section.

Cluster heat map analysis was conducted on the parameters at 7, 14 and 21 days, comprehensively visualizing the phenotypic traits, photosynthetic characteristics, antioxidant activity, nutritional components, yield and quality indicators of adzuki beans at different stages treated with photo-selective nets (**Figure 10**). The clustering heat map revealed that the five treatments were categorized into two major groups. The first cluster included RN and BN, where photosynthetic pigments (Chl a, Chl b, Chl a+b, and Car) and gas exchange parameters (Pn, Gs, and E) were significantly enhanced at 7, 14, and 21 days post-treatment. Chlorophyll fluorescence parameters (Fv/Fm, ΦPSII and qP) and antioxidant enzyme activities (POD, SOD and CAT) were up regulated to promote the substance accumulation and transformation, which ultimately enhanced yield and quality. The second cluster primarily comprised CK, YN and GN, which elevated H_2_O_2_, O_2_^-^, Ci, MDA, LA and NPQ levels. In contrast to the first cluster, the results suggested that spectral modification was the primary factor for clustering determinant.

### Correlation analysis

**Figure 11:**
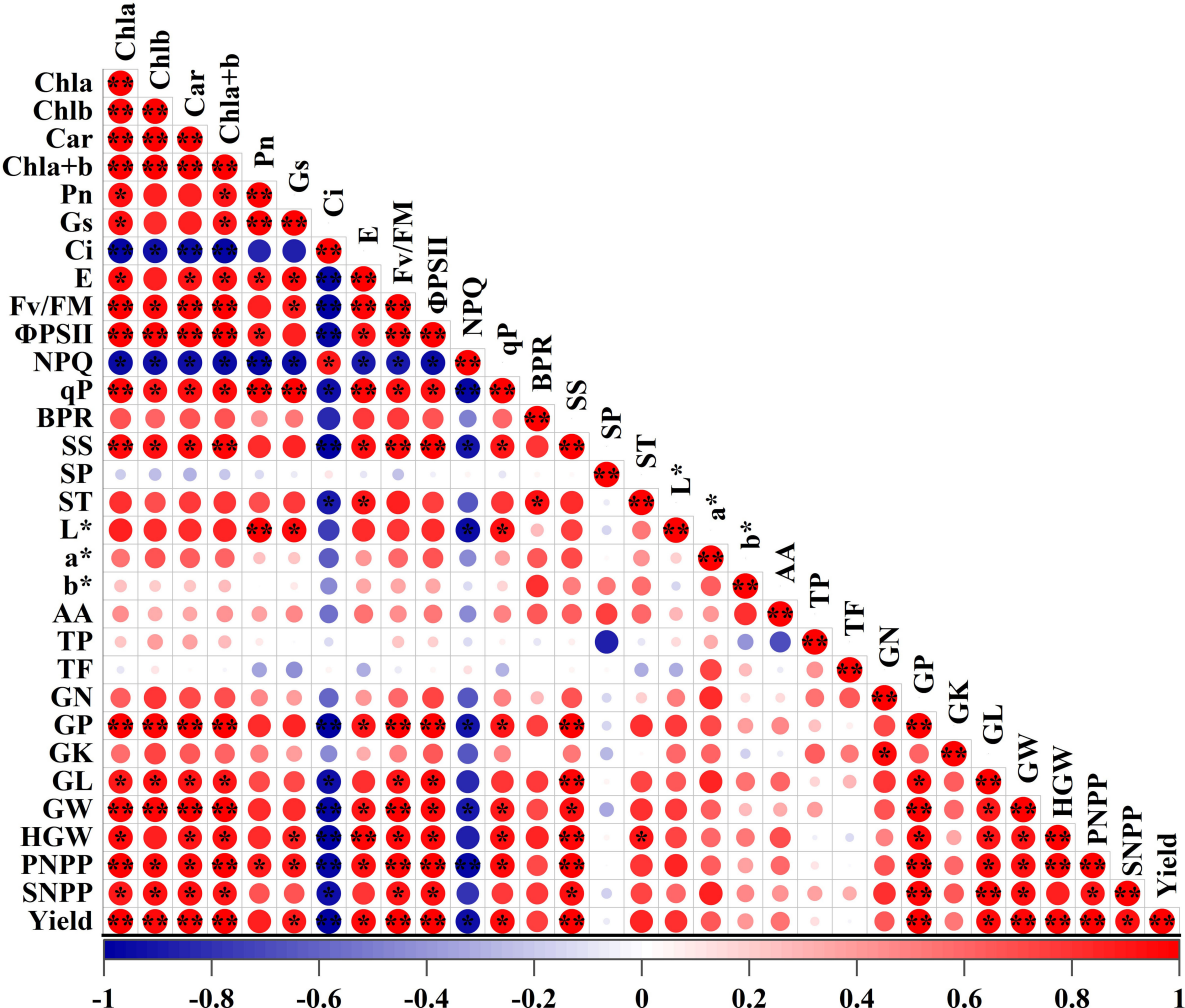
Correlation analysis between photosynthetic indices and yield quality traits in adzuki beans under photo-selective net treatments. Abbreviations of traits are listed in the abbreviations section * and * * were significant differences at P < 0.05 and P < 0.01, respectively.

Pearson’s correlation analysis was performed to examine the relationships between photosynthetic physiological indices (photosynthetic pigments, photosynthetic parameters, and chlorophyll fluorescence parameters) and yield-related quality traits of adzuki beans (**Figure 11**). The analysis demonstrated a highly significant positive correlation between chlorophyll content and yield (r^2^ > 0.90, P < 0.01), indicating that higher chlorophyll levels significantly correlated with greater yield under the experimental conditions. This can be attributed to the enhanced photosynthetic capacity driven by elevated pigment levels, which in turn promotes the biomass accumulation. A significant positive correlation was observed between Pn and pods per plant (PNPP) (r^2^ > 0.90, P <  0.05), suggesting that an increase in photosynthetic rate effectively enhances pod number per plant. Fv/Fm and ΦPSII also exhibited strong positive correlations with yield-related indices (Yield, PNPP, SNPP and HGW), indicating that optimal function of photosystem II enhances photosynthetic efficiency, thereby promoting grain yield improvement. Photosynthetic pigments and chlorophyll fluorescence parameters (Fv/Fm, ΦPSII, and qP) showed significant positive correlations with soluble sugar content in adzuki bean seeds, indicating that enhanced photosynthesis significantly promotes soluble sugar accumulation in seeds. Overall, significant positive relationships were observed among photosynthetic pigments, photosynthetic efficiency, chlorophyll fluorescence parameters, and grain yield. These findings indicate that photosynthetic efficiency plays a pivotal role in yield enhancement.

## Discussion

Photo-selective nets are recognized for their ability to mitigate excessive solar radiation, reduce leaf temperature and evaporative demand, regulate crop-level microclimate conditions, and enhance overall crop health and growth. They also alter the spectral composition of incident radiation, thereby inducing the metabolic adjustments in the photosynthetic system (Sabino et al., 2016), which likely contributed to the improved plant growth traits observed in this study. Analysis of the growth parameters throughout the experiment period revealed that the red photo-selective net significantly enhanced plant height, leaf area, above-ground dry weight, and total dry weight of adzuki beans compared to the control, which aligns with the findings reported in coffee trees (Henrique et al., 2011). The increased plant height under shade nets may be attributed to the enhanced light competition under low-light conditions, prompting plants to grow taller by producing leaves with greater length and breadth. In contrast, the blue photo-selective net treatment reduced the plant height of adzuki beans (**Table 2**), a result consistent with findings reported for *Myrsine africana* (Coles et al., 2021). However, plants under BN treatment exhibited significantly greater stem diameter, root dry weight, and root-shoot ratio than those under CK, and RN, GN, and YN treatments, These findings are consistent with those in soybean (Ma et al., 2018). This phenomenon can be attributed to cryptochromes (e.g., CRY1), which function as blue/UV-A photoreceptors (320–450 nm), and phytochromes (e.g., phyB), the primary red/far-red photoreceptors (600–750 nm)—the core photoreceptors that mediate light signal transduction to regulate plant morphogenesis (Ninu et al., 1999; Chen et al., 2021). For blue light (BN treatment), CRY1 is activated upon photon absorption and subsequently interacts with downstream signaling factors (e.g., the ubiquitin ligase COP1). This interaction represses hypocotyl elongation while promoting radial stem growth and root system development—consistent with the 8.15% higher stem diameter, 21.62% higher root dry weight, and 23.53% higher root-to-shoot ratio observed under BN treatment (Wu and Spalding, 2007; Peng et al., 2021). In contrast, red light (RN treatment) primarily activates phyB: under moderate shading (≈15.5% shading for RN treatment), phyB transitions from the inactive Pr form to the active Pfr form. The active Pfr form translocates into the nucleus, where it upregulates the expression of cell elongation-related genes (e.g., EXPANSINs, which encode cell wall-loosening proteins). This molecular regulation directly contributed to the increased plant height (12.23%) and total dry weight (18.04%) under RN treatment—an adaptive response of adzuki bean to compete for light resources (Henrique et al., 2011; Li et al., 2021).

Light quality plays a crucial role in regulating the content of photosynthetic pigments in plants (Peng et al., 2021). Chlorophyll is fundamental to plant photosynthesis. Studies have shown that the chlorophyll content in eggplant (Di et al., 2021) and cucumber (Su N et al., 2014) plants under red light was significantly higher than that under blue light. In this study, the chlorophyll content of plants in RN and BN treatments was significantly higher than that in CK plants, consistent with the previous findings. This can be attributed to the specific activation of light-signaling pathways by red/blue light: red light activates phyB, and blue light activates CRY1, which transduce signals to the nucleus to upregulate the expression of chlorophyll biosynthesis-related genes (e.g., protochlorophyllide oxidoreductase, POR; chlorophyll synthase, CHLG) and photosystem II (PSII)-related genes (e.g., psbA, encoding the D1 protein, a key component of PSII) (Chen et al., 2021; Peng et al., 2021). This molecular mechanism explains why only RN and BN treatments increased chlorophyll (Chl a, Chl b) and carotenoid contents: yellow light (YN) and green light (GN) cannot efficiently activate phyB or CRY1, thus lacking the regulatory trigger for pigment synthesis (Devlin et al., 2007; Yu et al., 2017). The effect is therefore driven by light quality rather than quantity, as no similar responses were observed under YN and GN treatments.” Carotenoids function as antenna pigments and components of the photosystem, being essential for its assembly, light harvesting, and photoprotection (Carvalho et al., 2011). In this study, carotenoid concentrations were highest under red and blue light treatments and lowest under green light treatments. The impact of varying light qualities on carotenoid concentrations seems to be species-specific. Previous studies have shown that blue light enhances carotenoid concentration in lettuce (Goto et al., 2010) and spinach (Ohashi et al., 2007), while red light supplementation promotes carotenoid accumulation in tomatoes (Tinyane et al., 2013). Ilić et al. (2012) revealed that β-carotene levels in tomato fruits from open-field control and red photo-selective net treatments significantly exceeded those from black or blue net treatments, highlighting red nets’ potential in enhancing carotenoid biosynthesis. This might be because photosynthetic pigments can better absorb light in the wavelengths of blue light (~430 nm) and red light (~660 nm) regions. Therefore, the red net increased the chlorophyll content and light capture ability of leaves (Devlin et al., 2007), thereby promoting the activity of photosynthetic pigments and enhancing carbon assimilation ability (Yu et al., 2017).

**Figure 12:**
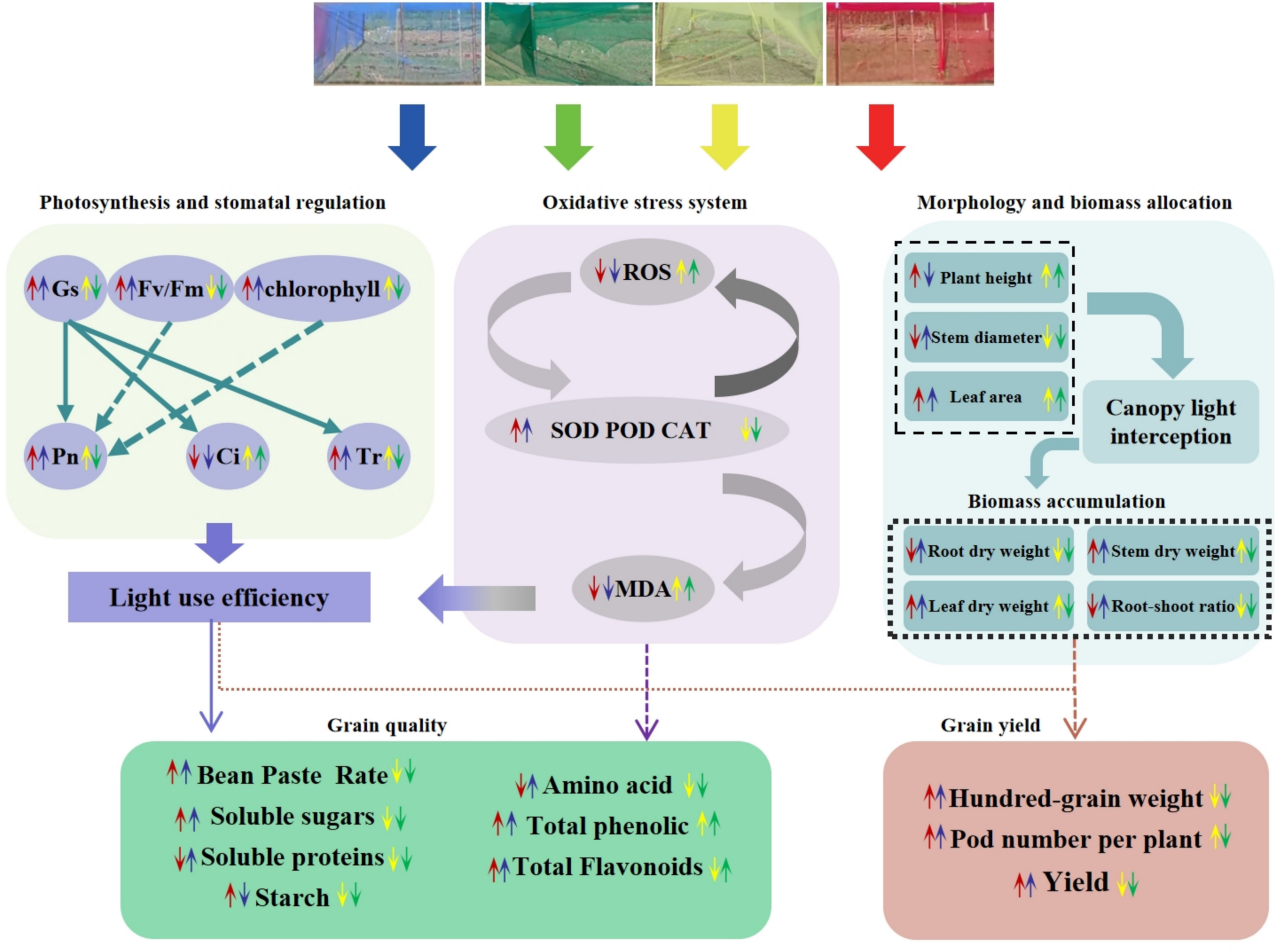
The mechanism of different photo-selective nets on the phenotypic traits, photosynthetic indicators, antioxidant capacity, yield and quality of adzuki beans.

Alterations in photosynthetic pigment content influence leaf photosynthesis, leading to changes in Pn, Gs, Ci and Tr. Previous studies have indicated that red light diminishes the net photosynthetic rate in rice and wheat (Yano and Fujiwara, 2012). Similarly, Ren et al. (2023a) reported that rice under blue light exhibited higher Pn values than under red light, while red light promoted higher Gs, Ci, and Tr values—these findings are inconsistent with the present study. In this experiment, RN-treated plants showed significantly higher Pn than other treatments, likely because higher Pn rates consumed more CO_2_, thereby reducing leaf Ci concentration. It is evident that photosynthetic rates display divergent responses to spectral-selective nets among different crops. These variations may stem from species-specific spectral absorption differences, coupled with multiple confounding factors including temperature, relative humidity, irrigation regimes, fertilization practices, and crop management protocols. The effects of comprehensive environmental factors on the morphogenetic characteristics of adzuki bean need to be further studied. Diurnal variation analysis of photosynthetic parameters (**Figure 6**) revealed that RN and BN coverings mitigated midday photosynthetic inhibition. This may be attributed to RN and BN mitigating midday photosynthetic inhibition by alleviating high-temperature stress and stomatal closure (Raveh et al., 2003). Chlorophyll fluorescence parameters serve as a powerful tool for evaluating crop photosynthetic performance and have been widely used to monitor photosynthetic efficiency in diverse crops (Guimarães et al., 2022). In this study, RN and BN treatments significantly enhanced Fv/Fm, ΦPSII, and qP compared to CK, while GN treatment caused significant reductions in Fv/Fm and increases in NPQ relative to other treatments. This indicated that the leaves under RN and BN treatments allocated more absorbed energy to the photochemical reactions, whereas GN treatment induced drastic reductions in adzuki bean photosynthesis and enhanced heat dissipation in LHCII. Photo-selective net treatments induced interactive effects on photosynthetic parameters. Under RN and BN, adzuki bean leaves exhibited increased thickness and compact palisade tissue arrangement, which promoted photosynthetic pigment accumulation. Concomitantly, enhanced Gs and Tr with reduced Ci but improved carbon assimilation, thereby elevating the maximum photochemical efficiency (Fv/Fm) of PSII under dark adaptation (**Figure 12**).

Malondialdehyde in leaves serves primarily as a biomarker for oxidative stress and membrane lipid peroxidation, directly reflecting the degree of cellular membrane damage. SOD, POD and CAT are key constituents of the antioxidant defense system, functioning to scavenge oxygen free radicals produced under stress conditions and thereby providing cellular protection against oxidative damage. This mechanism delays organismal senescence and cell death. Numerous studies have demonstrated that an increase in reactive oxygen species (ROS) under biotic and abiotic stress in plants is accompanied by the upregulated antioxidant enzyme activities. In this study, the leaves of adzuki beans under BN and RN conditions exhibited lower levels of O_2_^-^, H_2_O_2_, and MDA, while the activities of SOD, POD and CAT were significantly higher than those under other photo-selective net treatments (**Figure 8**). This suggests that BN and RN treatments effectively augment the antioxidant capacity of adzuki bean plants and delay senescence. This finding is consistent with the effect of light quality on green onion reported by Gao et al. (2021). However, Fan et al. (2022) revealed that red-blue light combination treatment promotes plant height, leaf number, leaf length, and leaf area in *Dendrobium officinale*, while enhancing SOD and POD activities, reducing MDA content, protecting cells from ROS damage, and promoting photosynthetic pigment accumulation. This study has not investigated the effects of combined lights on the antioxidant capacity of adzuki beans, and future studies will focus on this aspect.

Light conditions significantly influence crop yield and quality (Fu et al., 2017). Studies have shown that red net treatment has significantly improved the total yield and quality indicators of tomato fruits (Ilić et al., 2012, 2015). Son and Oh (2013) demonstrated that the red-weighted light spectrum improved the yield of lettuce. Tinyane et al. (2013) observed that red net treatment significantly increased the harvest yield of sweet peppers and tomatoes, which were consistent with the present study. The enhanced yield under shading conditions can be attributed to the increased plant height, leaf area, and above ground biomass. In this study, RN treatment significantly enhanced plant height, leaf area, total biomass, and photosynthetic pigment content, leading to elevated net photosynthetic rate and subsequent yield improvement (**Figure 12**). Photo-selective nets differentially impact the quality and nutritional composition of adzuki bean seeds. Zhang et al. (2010) reported that blue light cultivation increased soluble protein content in sweet potato leaves and pea sprouts. In this study, BN treatment significantly increased soluble protein and amino acid contents in adzuki bean plants compared to other treatments (**Table 3**). This may be attributed to the fact that protein synthesis, as a high-energy process for large molecules, benefits from the higher quantum energy of blue light, thus promoting protein biosynthesis (Zhang et al., 2010). The chlorophyll and soluble sugar contents of the leaves grown under the red led were relatively high (Tadda et al., 2023), which was consistent with the results of this study. This might be attributed to the higher photosynthetic rate under red light, which promotes the accumulation rather than consumption of photosynthetic products (Su J et al., 2014), thereby facilitating the biosynthesis of soluble sugars and starches. Photo-selective nets also exert significant effects on secondary metabolite accumulation. Ding et al. (2023) reported that blue light treatment maximized ascorbic acid (AsA), total phenols (TP), and total flavonoids (TF) in *Toona sinensis* sprouts, accompanied by enhanced DPPH and FRAP antioxidant activities. This study revealed that photo-selective nets differentially enhanced total phenol and flavonoid contents in adzuki bean grains, with RN and GN treatments achieving the highest levels, respectively. These discrepancies may stem from species-specific responses to photo-selective nets and the distinct microenvironmental conditions that they create.

## Conclusions

This study shows that the red and blue spectra can effectively regulate plant growth, photosynthetic efficiency and grain quality of adzuki beans. RN treatment significantly increased the plant height, total biomass and photosynthetic pigment content of the plants, resulting in a higher net photosynthetic rate and yield. BN treatment promoted the development of roots and stems and enhanced the contents of soluble proteins and amino acids. Both RN and BN treatments can improve the activities of antioxidant enzymes, reduce the accumulation of ROS, and alleviate photo-oxidative stress. Hence, red and blue photo-selective nets are recommended for application in adzuki bean cultivation and production to enhance photosynthetic physiological indices, thereby achieving the dual objectives of yield enhancement and quality improvement, and offering a novel theoretical basis for light environment optimization in leguminous crop cultivation.

## Data Availability

The raw data supporting the conclusions of this article will be made available by the authors, without undue reservation.

## Glossary

a*: Chromaticity coordinate (green-red)
AA: Amino acids content
b*: Chromaticity coordinate (blue-yellow)
BPR: Bean Paste Rate
Car: Carotenoid
CAT: Catalase
Chl a+b: Chlorophyll a+b
Chla: Chlorophyll a
Chlb: Chlorophyll b
Ci: Intercellular CO_2_ concentration
E: Transpiration rate, Fv/FM, Maximum photochemical efficiency
GK: Potassium content in grain
GL: Grain length
GN: Nitrogen content in grain
GP: Phosphorus content in grain
Gs: Stomatal conductance
GW: Grain width
H_2_O_2_: Hydrogen peroxide
HGW: Hundred-grain weight
L*: Lightness
LA: Leaf area
LADW: Leaf DW
LE: Lower epidermis
LK: Potassium content in leaves
LN: Nitrogen content in leaves
LP: Phosphorus content in leaves
MDA: Malondialdehyde
NPQ: Non-photochemical quenching coefficient
O_2_^-^: Superoxide anion radical
PH: Plant height
Pn: Net photosynthetic rate
PNPP: Pod number per plant
POD: Peroxidase
PP: Palisade parenchyma
qP: Photochemical quenching coefficient
RDW: Root DW
RK: Potassium content in root
RN: Nitrogen content in the root
RP: Phosphorus content in root
RPST: Ratio of palisade to spongy tissue
RSR: Root-shoot ratio
SD: Stem diameter
SDW: Stem DW
SK: Potassium content in stem
SN: Nitrogen content in stem
SNPP: Seed number per pod
SOD: Superoxide dismutase
SP: Spongy parenchyma
SP: Soluble proteins content
SP: Phosphorus content in stem
SS: Soluble sugars content
ST: Starch content
TDW: Total DW
TF: Total flavonoid content
TP: Total phenolic content
TT: Total thickness
UE: Upper epidermis
Yield: Yield
ΦPSII: Actual photochemical efficiency.
